# Risk Stratification and Strategies Towards Front-Line Therapy of EGFR-Mutant NSCLC: A Narrative Review

**DOI:** 10.3390/cancers18142285

**Published:** 2026-07-16

**Authors:** Kyle Taing, Hei Yeung Lam, Robert Hsu

**Affiliations:** 1 Department of Internal Medicine, Huntington Hospital, Pasadena, CA 91105, USA; kyle.taing2@huntingtonhealth.org; 2 Keck School of Medicine, University of Southern California, Los Angeles, CA 90033, USA; heiyeunl@usc.edu; 3 Division of Medical Oncology, Department of Medicine, University of Southern California, Los Angeles, CA 90033, USA

**Keywords:** EGFR-mutant NSCLC, osimertinib, TP53, RB1, CNS metastasis, leptomeningeal disease, ctDNA

## Abstract

**Background/Objectives:** Epidermal growth factor receptor (*EGFR*)-mutant non-small cell lung cancer (NSCLC) has undergone rapid therapeutic evolution. However, heterogeneous outcomes persist, driven by mutations, central nervous system (CNS) involvement, and dynamic tumor burden reflected in part by circulating tumor DNA (ctDNA). As such, this review aims to summarize the most recent risk stratification frameworks in treating *EGFR*-mutant NSCLC, evaluate evidence supporting treatment intensification strategies and managing adverse effects, and explore the evolving role of ctDNA in guiding personalized therapy. **Methods:** A comprehensive literature search was conducted using major medical databases with a focus on key relevant studies on the workup and management of *EGFR*-mutant NSCLC. All authors reviewed the literature, assessed study quality, and interpreted the results from each study. **Results:** Molecular co-alterations, such as *TP53* and *RB1*, as well as central nervous system (CNS) involvement, are consistently associated with inferior outcomes, supporting consideration of upfront treatment intensification. Combination strategies, including osimertinib plus chemotherapy or amivantamab-based regimens, demonstrate improved progression-free survival and delayed CNS progression when compared against osimertinib monotherapy. Intensification, however, is associated with a higher risk of increased toxicity, including dermatologic adverse events and infusion-related reactions. Finally, the utilization of circulating tumor DNA (ctDNA) has emerged as a strong prognostic marker, with ongoing trials investigating its predictive role for both escalation and de-escalation of therapy. **Conclusions:** The treatment paradigm for *EGFR*-mutant NSCLC is gradually evolving beyond first-line osimertinib to include a more integrated approach that considers molecular features, CNS involvement, and early ctDNA response. Although intensified regimens offer meaningful efficacy gains for high-risk patients, proactive toxicity management is essential to preserving quality of life. ctDNA-guided strategies represent a new and promising frontier for escalation and de-escalation of therapy, with results from ongoing trials poised to further refine personalized treatment algorithms.

## Simple Summary

For a long time, osimertinib was the first-line treatment for patients with non-small cell lung cancer (NSCLC) caused by *EGFR* mutations. However, when considering osimertinib versus combination therapy, a variety of factors such as additional genetic mutations, presence of metastasis to the central nervous system, and overall tumor burden should be considered. This review examines how patients are classified into higher- or lower-risk groups to better personalize treatment. Namely, patients with certain genetic mutations or brain involvement may benefit from starting with a stronger combination treatment, such as adding chemotherapy or other targeted drugs, rather than using osimertinib alone. We also discuss the role of circulating tumor DNA, which may assist in monitoring the progression of NSCLC and deciding when to increase or decrease treatment intensity. In summarizing the current state of *EGFR*-mutant NSCLC, this review aims to guide ongoing research in refining more personalized treatment plans.

## 1. Introduction

Non-small cell lung cancer (NSCLC) remains the leading cause of cancer-related mortality worldwide, accounting for approximately 85% of all lung cancer diagnoses [1]. Mutations in the epidermal growth factor receptor (*EGFR*) gene can be detected across all histologic subtypes of NSCLC, with the highest prevalence noted within the adenocarcinoma subtype. The EGFR gene specifically encodes a receptor tyrosine kinase that regulates cellular proliferation, angiogenesis, apoptosis, and migration [2]. The most clinically frequent *EGFR* mutations include the exon 19 deletions (del19) as well as the L858R point mutation in exon 21, which together account for 85 to 90% of all *EGFR* mutations [3]. Moreover, these mutations account for approximately 10 to 15% of NSCLC cases in Western populations and up to 40–50% in East Asian patients, with a higher prevalence among individuals who never smoked, women, and patients with a history of adenocarcinoma [4].

The panel of *EGFR*-specific tyrosine kinase inhibitors (TKIs) includes the first-generation class, such as gefitinib [5] and erlotinib [6], which are reversible inhibitors that select the target EGFR, and the second-generation TKIs, such as afatinib, which are irreversible pan-ErbB family inhibitors with activity against EGFR/HER1, HER2, and HER4 [7]. The introduction of these TKIs has previously resulted in marked improvements in progression-free survival (PFS) and overall quality of life compared to standard platinum-based chemotherapy. This firmly established *EGFR* inhibition as the standard initial approach for patients with advanced, unresectable NSCLC harboring activating *EGFR* mutations, though since then, the use of *EGFR* inhibition has gone beyond advanced, unresectable NSCLC to adjuvant use in *EGFR*-mutant NSCLC after surgery per the ADUARA trial and after definitive chemotherapy and radiation per the LAURA trial, and it is being studied prior to surgery per the NeoADAURA study [8,9,10]. However, acquired resistance— most commonly mediated by the T790M mutation—has been noted to blunt the durability of these responses [11,12,13]. As such, for over a decade, the standard first-line therapy in treating advanced *EGFR*-mutant NSCLC has evolved to include osimertinib, an oral third-generation irreversible (but selective) EGFR TKI with activity against both sensitizing *EGFR* mutations and T790M [14]. One landmark study, the AURA3 trial, demonstrated improved median PFS (10.1 months versus 4.4 months) with osimertinib when compared against platinum-pemetrexed chemotherapy (hazard ratio [HR] 0.30, 95% confidence interval [CI] 0.23–0.41, *p* < 0.001) in patients with T790M-positive disease following progression on earlier-generation TKIs [15]. Subsequently, in the phase III FLAURA trial, osimertinib significantly improved median PFS (18.9 versus 10.2 months, HR 0.46, 95% CI 0.37–0.57, *p* < 0.001) when compared to a standard EGFR TKI such as gefitinib or erlotinib, while also demonstrating superior CNS penetration and a more favorable toxicity profile [16].

Despite the advent of osimertinib, however, a considerable proportion of patients continue to experience disease progression and ultimately die of lung cancer because of resistance to treatment. Multiple mechanisms of osimertinib resistance have been recognized, most prominent of them being alterations in the *MET* gene [17] and *EGFR* pathways [18], along with on-target mutations such as C797X [19], yet we currently do not know the mechanisms of resistance for over half of the patient population. As such, research focusing on improving efficacy outcomes in these patients remains ongoing, especially for subgroups of patients with poor prognosis. In particular, two landmark phase III randomized trials (FLAURA2 and MARIPOSA) have focused on the addition of chemotherapy or novel biologic agents to EGFR inhibition.

The FLAURA2 study is a randomized phase III trial (n = 557) that compared first-line osimertinib monotherapy against a combination regimen consisting of osimertinib plus platinum-pemetrexed chemotherapy in patients with advanced *EGFR*-mutated NSCLC. The primary endpoint of PFS was significantly longer with the combination regimen (median 25.5 vs. 16.7 months, HR 0.62, 95% CI 0.49–0.79, *p* < 0.001), with particular benefit in patients with L858R mutations (24.7 vs. 13.9 months) [20]. Benefit in overall survival was also demonstrated, with a median OS of 47.5 vs. 37.6 months (HR 0.77, 95% CI 0.61–0.96, *p* = 0.02) in patients on the combination regimen as opposed to those on monotherapy [21]. Similarly, the MARIPOSA study is a randomized phase III trial that compared the effect of osimertinib against a combination therapy of amivantamab (a bispecific antibody against EGFR and MET receptor) and lazertinib (a third-generation TKI) in treatment-naive patients with advanced *EGFR*-mutated NSCLC. The primary endpoint of PFS was significantly longer with amivantamab–lazertinib (median 23.7 vs. 16.6 months, HR 0.70, 95% CI 0.58–0.85, *p* < 0.001) when compared against osimertinib alone [22]. Again, benefit in overall survival was also demonstrated, with a 3-year OS of 60% in the combination regimen against 51% in the monotherapy arm (HR 0.75, 95% CI 0.61–0.92, *p* = 0.005), as well as projected OS advantage exceeding 12 months [23]. Notably, a more recent study utilized the data from MARIPOSA to demonstrate a comparable median PFS between lazertinib monotherapy and osimertinib monotherapy (18.5 vs. 16.6 months, HR 0.98, 95% CI 0.79–1.22, *p* = 0.86) in previously untreated *EGFR*-mutant advanced NSCLC [24].

More recently, the COMPEL trial evaluated patients with *EGFR*-mutated advanced NSCLC following non-central nervous system (CNS) progression on first-line osimertinib. Median PFS was significantly longer (8.4 vs. 4.4 months, HR 0.43, 95% CI 5.7–11.8 months) with osimertinib continuation plus chemotherapy, and CNS PFS was also longer with osimertinib continuation in patients without baseline CNS metastases (HR 0.56, 95% CI 0.27–1.13). Median OS also trended favorably at 15.9 vs. 9.8 months (HR 0.71, 95% CI 0.42–1.23) [25].

These landmark studies, namely FLAURA2 and MARIPOSA, have been paramount in shifting the therapeutic framework away from a uniform first-line approach (consisting solely of a third-generation TKI) towards one of treatment intensification. However, although the strategies employed by these two studies were shown to prolong PFS and delay CNS progression, they were also associated with an increased risk of toxicity. Namely, the most commonly reported adverse events (AEs) for the FLAURA2 regimen included hematologic and gastrointestinal toxicity; whereas dermatologic toxicity, venous thromboembolisms, and infusion-related reactions were the key AEs noted in the MARIPOSA regimen [21,22]. As such, these developments open new perspectives and possibilities in the treatment algorithms for *EGFR*-mutant NSCLC, namely by raising critical questions regarding optimal patient selection, risk stratification, and the balance between efficacy and tolerability in the first-line setting. In light of the rapidly evolving therapeutic landscape of *EGFR*-mutant NSCLC, we aim to provide a comprehensive clinical overview in evaluating patients with *EGFR*-mutant NSCLC, namely in regard to risk stratification as well as updates in combination regimens. This will entail a discussion on risk factors such as co-mutations and CNS involvement, key takeaways from recent landmark trials in managing side effects from various combination regimens, and future directions such as the use of ctDNA in guiding treatment intensification.

## 2. Search Strategy

The PubMed and Google Scholar databases, with default settings and no restrictions, were used to search the literature in this review. “EGFR” and each of its aliases (“ErbB-1” and “HER1”) were used in combination with “NSCLC” to search the literature specific to the therapy frameworks in managing *EGFR*-mutant NSCLC.

## 3. Risk Stratification

Despite the transformative impact of third-generation EGFR TKIs (namely osimertinib), outcomes among patients with *EGFR*-mutant NSCLC remain heterogeneous. This variability reflects underlying molecular complexity as well as patterns of metastatic spread, particularly central nervous system (CNS) involvement. Therefore, risk stratification is increasingly critical to identify patients who may benefit from treatment intensification beyond EGFR TKI monotherapy and to balance anticipated efficacy gains against cumulative toxicity.

### 3.1. Molecular Risk Factors

Tumor protein p53 (*TP53*) is one of the most frequently co-altered genes in *EGFR*-mutant NSCLC, occurring in approximately 55–65% of cases [26]. Given that TP53 encodes a tumor suppressor protein that regulates cell-cycle arrest and apoptosis, a loss-of-function mutation in this gene promotes chromosomal instability and an inherently higher risk of developing malignancy. This mutation consistently predicts worse outcomes in EGFR-mutant NSCLC across multiple meta-analyses and cohort studies. By using hazard ratio (HR) analysis, one systematic review demonstrated that TP53 mutations were significantly associated with both shorter PFS (HR = 1.51, 95% CI 1.33–1.71, *p* < 0.001) as well as shorter overall survival (OS) (HR = 1.64, 95% CI 1.33–2.02, *p* < 0.001) compared to the absence of a TP53 mutation. Furthermore, TP53 mutations were not associated with the objective response rate (ORR) of EGFR-TKI treatment (odds ratio 0.91, 95% CI 0.69–1.21, *p* = 0.529), suggesting that TP53 mutations may be associated with primary resistance to EGFR-TKIs [26].

Importantly, the prognostic impact of TP53 co-mutations is not uniform and depends on the specific mutation type and functional domain involved. A recent study demonstrated that TP53 mutations involving the DNA-binding domain (DBD) were associated with significantly shorter PFS compared to non-DBD TP53 mutations and TP53 wild-type, particularly in patients treated with first- or second-generation EGFR-TKIs [27]. TP53 mutations can be further classified as disruptive versus non-disruptive and by exon location, demonstrating that exon 8 mutations and pathogenic/disruptive mutations were associated with the worst PFS and OS outcomes [28]. Beyond structural and domain-based classifications, TP53 co-mutations can also be categorized by function, such as loss-of-function mutations and gain-of-function (GOF) mutations [29]. Specifically, TP53 GOF mutations activate TNF-α–NF-κB signaling, which can subsequently lead to osimertinib resistance [30]. One recent study demonstrated TP53 GOF co-mutations in EGFR-mutant NSCLC were associated with significantly shorter PFS on first-line osimertinib compared to both TP53 wild-type (median 12.0 vs. 31.4 months, *p* = 0.0016) and TP53 non-GOF mutations (median 12.0 vs. 21.9 months, *p* = 0.038) [31]. These findings underscore that GOF mutations define a biologically distinct subtype characterized by early resistance to osimertinib. Moreover, this suggests that the prognostic impact of TP53 co-mutations at large is likely modulated by the specific functional consequences of each mutation, further supporting the need for more nuanced and specific TP53 classification beyond a binary mutant versus wild-type status.

Furthermore, TP53 alterations were associated with faster acquisition of resistance mutations (including T790M) and higher mutational burdens, suggesting that TP53 facilitates resistance evolution independent of the specific resistance mechanism [32]. Collectively, these findings not only highlight the molecular and functional complexity of TP53 mutations as a limitation of many retrospective studies that treat TP53 status as a binary variable but also underscore the need for domain-specific and exon-level TP53 classification in future prospective trials to refine risk stratification.

The retinoblastoma 1 (*RB1*) gene also plays a critical role in cell-cycle regulation, specifically via inhibition of E2F transcription factors to control G1-S phase transition [33]. Although concurrent *RB1* and *TP53* mutations represent approximately 5% of *EGFR*-mutant NSCLC, they are not only associated with a similarly poor prognosis, but they also represent a unique risk of histologic transformation to small-cell lung cancer (SCLC). One study analyzing tumor samples and cell lines derived from *EGFR*-mutant patients revealed that *RB1* is lost in 100% of SCLC-transformed cases, but rarely in those that remain in NSCLC [34]. In another study, patients with *EGFR/TP53/RB1*-mutant NSCLC had a significantly shorter time to EGFR-TKI discontinuation (9.5 months) when compared against *EGFR/TP53* (12.3 months) and *EGFR*-mutant only (36.6 months) malignancy (*p* < 0.001). This study also demonstrated that 18% of patients with the triple-mutant cancer had eventual SCLC transformation [35]. Furthermore, a more recent study in patients with concurrent *TP53* and *RB1* alterations (n = 11) demonstrated that the addition of platinum and etoposide to osimertinib resulted in promising activity with an ORR of 82%, a median PFS of 15.6 months, and a median OS of 37.9 months [36]. These findings collectively suggest that EGFR-TKI monotherapy alone may not appropriately treat patients with this triple-mutation.

Given the uniquely poor prognosis in NSCLC patients with concomitant mutants, recent studies have attempted to compare combination therapy against TKI monotherapy in addressing these concurrent mutations. One retrospective analysis focusing on NSCLC with concomitant *EGFR* and *TP53* mutations (n = 124) demonstrated a median PFS of 18.0 months (95% CI 12.1–23.9 months) when treated with EGFR-TKIs combined with antiangiogenic drugs or chemotherapy. This is a significantly longer increase (*p* < 0.001) in median PFS when compared to that of 7.0 months (95% CI 6.1–7.9 months) in patients treated with TKI monotherapy [37]. Another *EGFR/TP53* NSCLC study (n = 95) demonstrated a significant improvement in ORR (55.9% vs. 34.4%, *p* = 0.042) as well as median time to progression (TTP) (16.1 vs. 11.1 months, *p* = 0.002) in patients who received EGFR-TKI in combination with chemotherapy when compared to EGFR-TKI alone [38]. Furthermore, a secondary analysis of the MARIPOSA trial demonstrated that the amivantamab–lazertinib regimen improved median PFS (18.2 vs. 12.9 months, HR 0.65, 95% CI of 0.48–0.87, *p* < 0.001) when compared against osimertinib monotherapy for patients with *TP53* co-mutations [39]. Finally, a more recent TOP trial demonstrated that in patients with *EGFR/TP53* co-mutated advanced NSCLC, the median PFS is doubled in the combination therapy arm consisting of osimertinib and carboplatin/pemetrexed (34.0 vs. 15.6 months, HR 0.44, 95% CI of 0.32–0.60, *p* < 0.001) when compared against osimertinib monotherapy [40]. Although research focusing on treating *RB1* co-mutations and triple-mutation disease remains ongoing, these *TP53* studies underscore the need for early consideration of alternative therapies, namely combining EGFR-TKIs with systemic chemotherapy.

*MET* amplification represents a very common *EGFR*-independent mechanism of acquired resistance to third-generation EGFR-TKIs, in which the *MET* proto-oncogene encodes a transmembrane receptor tyrosine kinase that activates downstream PI3K/AKT, JAK/STAT, and RAS/MAPK/ERK signaling pathways independently of EGFR, thereby bypassing EGFR inhibition [41]. *MET* amplification can also occur as a de novo co-alteration in treatment-naive *EGFR*-mutant NSCLC, contributing to primary resistance. Dual inhibition of *EGFR* and *MET* has emerged as a rational therapeutic strategy, as studied via the MARIPOSA trial with amivantamab [22]. More recently, however, the phase III SACHI trial further demonstrated that savolitinib plus osimertinib significantly improved PFS compared to chemotherapy in *MET*-amplified, *EGFR*-mutant NSCLC (mPFS 11.1 vs. 4.4 months, HR 0.33, *p* < 0.0001) [42]. Moreover, the INSIGHT 2 trial showed that tepotinib plus osimertinib achieved an ORR of 50% in patients with confirmed *MET* amplification [43]. These findings highlight the importance of post-progression molecular testing, including assessment for *MET* amplification, to guide subsequent therapy selection.

Additional genetic alterations, including but not limited to *PIK3CA/KRAS* mutations as well as *PTEN* inactivation, are also important considerations in treating *EGFR*-mutant NSCLC. Mutations in *PIK3CA*, a gene that regulates cell growth and survival via mTOR activation, occur in approximately 8% of these malignancies [44]. Co-alteration of *TP53* and *PIK3CA* was associated with a poor prognostic effect, as demonstrated by significantly inferior median OS (21.9 versus 39.5 months, *p* = 0.046) and median time-to-treatment discontinuation, mTTD (13.7 versus 25.8 months, *p* = 0.071) when compared against wild-type [45]. Similarly, inactivation of *PTEN*, a tumor suppressor gene that promotes apoptosis (also via the mTOR pathway), is seen in approximately 5–12% of *EGFR*-mutant NSCLC [46]. *PTEN* mutations were also associated with a significantly shorter median PFS (2.6 months vs. 10.3 months, *p* = 0.001) on third-generation EGFR TKIs [47]. Another study demonstrated that in *EGFR*-dependent cells, *PTEN* loss partially uncouples mutant EGFR from downstream signaling and activates EGFR, which may explain the poor prognosis associated with this mutation [48]. Finally, one study (n = 124) notably demonstrated that the frequency of *PIK3CA/PTEN/RAS* mutations in patients with *EGFR*-mutant NSCLC was 61.7%. Specifically, *PIK3CA/PTEN/RAS* co-mutations were associated with worse OS than in patients with *RAS/PIK3CA/PTEN* mutations (53.8 months vs. 27.4 months). However, there was no significant difference in EGFR TKI response, suggesting that a combination approach is merited to help overcome inferior OS outcomes [49].

*EGFR* and *KRAS* mutations have historically been considered mutually exclusive driver events [50]. However, concurrent *EGFR* and *KRAS* mutations have previously been identified, though reported frequencies vary substantially depending on detection methodology and cohort composition. In one early study of NSCLC cases using targeted sequencing, 1.1% harbored concurrent *EGFR* and *KRAS* mutations [51]. However, a larger NGS-based landscape study of EGFR-positive NSCLC identified concomitant driver alterations in 3.1% of cases, with *KRAS* being the most frequent co-occurring driver (53.9% of all concomitant driver cases), followed by *ERBB2* (24.3%) and MET (16.5%); of note, among *EGFR/KRAS* concomitant mutations, there was a greater frequency of hyperexchange mutations, such as (KRAS G13D and G12X variants), compared to single driver mutations [52]. More specifically, when co-occurring, these *KRAS* mutations may exist in either cis (within the same cancer cell) or trans (in different subclonal populations) configurations, reflecting intratumoral molecular heterogeneity. NGS-based studies using variant allele frequency (VAF) analysis have provided some insight: in cases where *EGFR* mutations are detected at high VAF and *KRAS* at low VAF (or vice versa), a trans relationship is suggested [53]. Beyond *KRAS*, other *RAS* family genes (e.g., *NRAS*, *HRAS*) are rarely reported in EGFR-mutant NSCLC, and their clinical significance in this context remains poorly defined [54]. The question of whether concurrent *EGFR/KRAS* mutations represent true co-occurrence within the same cell versus intratumoral heterogeneity with distinct subclonal populations remains an active area of investigation, with implications for treatment selection and resistance monitoring.

Emerging evidence supports the classification of *EGFR*-mutant NSCLC with high programmed death ligand 1 (PD-L1) as a biologically and clinically distinct sub-population. PD-L1 is a known immune system suppressor protein that allows tumor cells to evade anti-tumor immunity. A recent analysis (n = 1488) confirmed that PD-L1 tumor proportion score (TPS) was the strongest predictor of very early progression on first-line osimertinib, surpassing even TP53 co-mutation status in predictive importance [55]. Furthermore, high PD-L1 expression in *EGFR*-mutant NSCLC was associated with a higher frequency of uncommon and complex *EGFR* mutations, and it also independently predicted shorter survival on multivariate analysis [56]. These findings suggest that high PD-L1 expression (specifically, a TPS of 50% or greater) in *EGFR*-mutant NSCLC identifies a high-risk group that may benefit from upfront treatment intensification with combination regimens rather than osimertinib monotherapy, and that PD-L1 testing should be incorporated into risk stratification frameworks alongside molecular co-alterations.

Table 1 below provides an overview of the molecular risk factors that should be considered when risk-stratifying patients with *EGFR*-mutant NSCLC. Overall, further research does need to be performed in comparing TKI monotherapy against combination therapy regimens, including chemotherapy. However, the above studies (including *TP53*, *RB1*, and the other less common mutations) have so far highlighted that the presence of these genetic alterations warrants consideration for escalation of therapy and highlights the importance of co-mutations when considering the heterogeneous biology of *EGFR*-mutant NSCLC.

### 3.2. Central Nervous System Involvement

CNS involvement represents one of the most clinically significant features in *EGFR*-mutant NSCLC, with up to 25–30% of patients presenting with brain metastasis at the time of diagnosis, and as many as 50–60% of patients developing intracranial disease as the NSCLC progresses [57]. This high incidence includes both parenchymal brain metastases, which are typically limited in number and size, as well as leptomeningeal disease (LMD), which is classically characterized by widespread dissemination within the cerebrospinal fluid. As such, LMD is associated with limited drug penetration and poor prognosis; patients with *EGFR* mutations (9.4%) also have a higher incidence of leptomeningeal disease compared to *EGFR* wild-type disease (1.7%) [58]. Both brain metastases and LMD represent a subset of *EGFR*-mutant NSCLC that has been historically difficult to manage due to the distinct tumor biology within the CNS. Such barriers include but are not limited to the blood–brain barrier, the brain–CSF barrier (with efflux pumps), and the tumor microenvironment [59]. Furthermore, earlier-generation TKIs were shown to have low CNS-to-plasma ratios, which led to incomplete suppression of intracranial metastases and higher rates of CNS progression [60]. Collectively, these various factors contribute to the CNS being recognized as a “sanctuary site,” a pharmacologic and biologic refuge for tumor cells that continues to be addressed by both landmark and ongoing studies as described below [61].

Osimertinib has emerged as the preferred first-line TKI due to superior CNS activity when compared against first-generation TKIs. In the FLAURA trial, osimertinib achieved a 91% CNS objective response rate (ORR) versus 68% with first-generation TKIs, with a median CNS PFS not reached versus 13.9 months (HR 0.48, 95% CI 0.26–0.86, *p* = 0.014) [62,63]. Another study recruited *EGFR* T790M-positive NSCLC patients with CNS metastasis who progressed on prior EGFR TKIs, which demonstrated promising intracranial ORR (55.0%) and survival benefit (median PFS of 7.6 months, median OS of 16.9 months) with a tolerable safety profile [64]. Moreover, the phase I BLOOM trial showed that osimertinib demonstrated meaningful therapeutic efficacy in the CNS and a manageable safety profile at 160 mg once daily in patients with *EGFR*-mutated NSCLC and LMD. The study specifically demonstrated a high ORR of 62%, improved neurological function in 57% of patients, and confirmed CSF tumor cell clearance in 28% of patients [65]. A more recent phase II study known as the ACHIEVE trial has attempted to emulate the findings demonstrated in BLOOM by using high-dose aumolertinib, another third-generation EGFR TKI [66]. As such, current practice generally favors initiation of osimertinib in such patients with CNS involvement, particularly for asymptomatic or minimally symptomatic disease [67].

Despite the high initial CNS response rate previously achieved with osimertinib, however, more recent data suggest that patients with baseline CNS metastases may benefit from escalation to combination strategies. For example, the FLAURA-2 trial demonstrated a longer PFS with osimertinib plus platinum-based chemotherapy when compared against osimertinib alone in patients with CNS metastases (24.9 vs. 13.8 months, HR 0.47, 95% CI 0.33–0.66) [20,68]. These pivotal findings were further supported by a meta-analysis of 24 randomized controlled trials involving 2682 patients, which suggested that the combination of third-generation TKIs in combination with systemic chemotherapy provided the most significant improvements in OS (HR 1.69, 95% CI 1.14–3.4) and PFS (HR 2.13, 95% CI 1.28–3.54) when compared against third-generation TKI alone [69]. These more recent studies suggest that CNS involvement in itself is a compelling risk factor that warrants intensification in *EGFR*-mutant NSCLC management. These findings also justify the need for a shift in treatment guideline recommendations towards early initiation of a combination regimen, consisting of both osimertinib along with platinum-based chemotherapy, in this specific patient population [70]. That being said, many metastatic *EGFR*-mutant patients with CNS involvement are not physically fit for combination therapy at the time of diagnosis, and consideration of studies in which patients can intensify therapy when becoming more fit is warranted.

**Figure 1:**
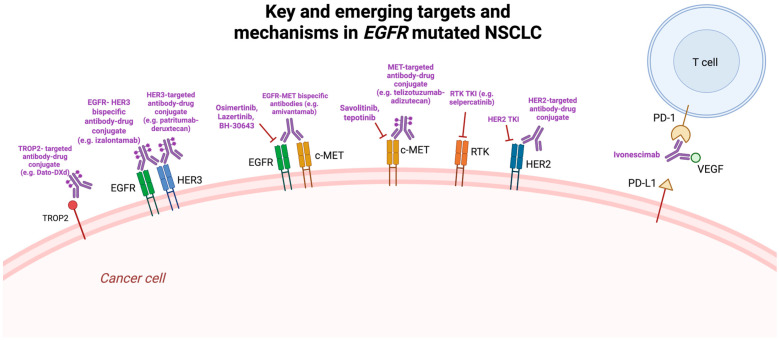
Key and emerging targets and mechanisms in *EGFR*-mutated NSCLC. This includes antibody–drug conjugates, including datopotomab deruxtecan, which is now FDA approved in *EGFR*-mutated NSCLC after disease progression with platinum-based chemotherapy and *EGFR* TKI, osimertinib in combination with chemotherapy per the FLAURA2 study, and amivantamab in combination with lazertinib per the MARIPOSA study. Emerging targets in clinical trial development include BH-30643, which is a new generation *EGFR* TKI that can overcome C797S acquired mutations, savolitinib, which is a MET TKI being used in combination with *EGFR* TKI as a way to overcome MET alterations in *EGFR*-mutated NSCLC, along with MET-targeted antibody–drug conjugates such as telisotzumab-adizutecan, HER3 antibody–drug conjugates such as izalontamab, and ivonescimab, a PD-1/VEGF bispecific antibody being studied in disease progression after *EGFR* TKI [22,71,72,73,74,75,76,77]. (EGFR = epidermal growth factor receptor, NSCLC = non-small cell lung cancer, TROP-2 = Trophoblast cell surface antigen-2, HER3 = Human Epidermal growth factor Receptor 3, MET = Mesenchymal-Epithelial Transition factor, RTK = Receptor Tyrosine Kinase, Human Epidermal growth factor Receptor 2, PD-1 = Programmed death-1, PD-L1 = Programmed death-Ligand 1, VEGF = Vascular Endothelial Growth Factor).

Beyond osimertinib, however, other combination regimens must be considered in therapy intensification, especially in patients with osimertinib-refractory disease. Namely, the MARIPOSA trial (via the amivantamab–lazertinib combination regimen) demonstrated similarly improved efficacy in patients with CNS metastasis when compared to osimertinib alone, including a significantly prolonged PFS, OS, and duration of response (DOR) while maintaining intracranial objective response rates [22]. These findings were further corroborated by a recent phase II study (n = 41) that demonstrated an ORR of 50% (95% CI 27–73%) and 33% (95% CI 15–57%) in *EGFR*-mutant patients with brain metastases and LMD, respectively. Furthermore, the brain metastasis cohort had a median PFS of 5.8 months (95% CI 3.6—not reached) and a median OS of 17.4 months (95% CI 15.4—not reached), whereas the LMD cohort had a median PFS of 7.8 months (95% CI 4.2–12.2) and a median OS of 14.4 months (8.9—not reached) [71]. This led to amivantamab + lazertinib being recognized as a first-line treatment option for *EGFR*-mutated NSCLC. Furthermore, there are additional studies currently underway to further explore other potential favorable regimens in treating *EGFR*-mutated NSCLC with CNS involvement, including savolitinib plus osimertinib (SAVANNAH trial, phase II) for *EGFR*-mutant NSCLC with *MET* alterations and disease progression, as well as datopotamab deruxtecan (Dato-DXd) (TROPION-Lung05 trial, phase II), which was recently FDA-approved for *EGFR*-mutant NSCLC in patients with disease progression after platinum-based chemotherapy and osimertinib [72,73]. In addition, there are other promising regimens such as Dato-DXd in combination with osimertinib per the ORCHARD study [74] and ongoing TROPION-Lung14 study [75], ivonescimab (a PD-1 and VEGF bispecific antibody) per the HARMONI-A study [76], and BH-30643 (a novel, orally available, non-covalent, macrocyclic inhibitor that could overcome C797S acquired mutations) [77]. Figure 1 below depicts key and emerging targets in treating *EGFR*-mutated NSCLC.

Importantly, the role of radiation therapy, while not the primary focus of systemic treatment intensification strategies, remains a critical component that should be considered within a multidisciplinary framework. One such radiation strategy is known as stereotactic radiation therapy, which is effective for limited symptomatic lesions, providing local control with low toxicity. One study showed that stereotactic radiation therapy, when used in combination with high-dose osimertinib, demonstrated meaningful intracranial activity in both *EGFR*-mutant NSCLC patients with brain metastases (disease control rate of 77.5%) as well as LMD (disease control rate of 92.5%) [64]. Furthermore, ongoing studies are also beginning to explore the possible utility that next-generation sequencing may provide in NSCLC management, specifically in further guiding management in treatment intensification and de-escalation [78,79]. Furthermore, ongoing studies are also beginning to explore the utility that genotyping may provide in treatment intensification and de-escalation, with one such method being tissue-based next-generation sequencing in patients with CNS involvement. Another robust method has involved the molecular profiling of cerebrospinal fluid (CSF), where a recent study identified patients who are at higher risk based on biomarker alterations on CSF analysis. Specifically, co-alterations in *CDK4*, *CDK6*, and *MYC* were associated with poorer outcomes in patients with *EGFR*-variant lung adenocarcinoma (with HR ranging from 2 to 2.5) [80].

Historically, osimertinib has significantly improved intracranial response rates and clinical outcomes in *EGFR*-mutant NSCLC patients with CNS involvement. However, a growing body of data supports early treatment intensification with combination strategies, including platinum-based chemotherapy or amivantamab–lazertinib, to optimize disease control as recommended by the most updated American Society of Clinical Oncology Living Guideline [67]. Concurrently, advances in radiation techniques and molecular profiling are further refining risk stratification and enabling more personalized therapeutic approaches. Overall, these evolving insights underscore a paradigm shift from reliance on TKI monotherapy toward a more aggressive and multimodal strategy to better address the biologically unique “sanctuary site.”

## 4. Managing Combination Regimen Side Effects

Despite the statistically and clinically significant outcomes demonstrated in the FLAURA2 and MARIPOSA trials, they are also associated with substantially increased toxicity burden when compared against osimertinib monotherapy. As such, each intensification strategy carries a fundamentally distinct adverse effect (AE) profile that has important implications for patient selection, monitoring, treatment, and supportive care. In the FLAURA2 trial, grade 3 or higher AEs of any cause were reported in 70% of the hybrid therapy arm as opposed to 34% of the monotherapy arm [21]. The dominant toxicity driver in the combination arm was myelosuppression (e.g., anemia, neutropenia, thrombocytopenia), consistent with the known effects of platinum-pemetrexed chemotherapy [81]. Gastrointestinal effects were the second most-reported AEs of the FLAURA2 trial, including but not limited to nausea, decreased appetite, diarrhea, and stomatitis. Other notable (albeit less commonly reported) adverse effects included fatigue, rash, ILD/pneumonitis, elevated liver transaminases, and peripheral edema. Adverse effects leading to discontinuation of any trial of treatment occurred in 54% of patients in the combination arm at the final analysis, compared against 7% in the osimertinib monotherapy arm.

Similarly, in the MARIPOSA trial, the cohort receiving the intensified treatment (amivantamab + lazertinib) experienced more AEs than the cohort on osimertinib alone. Specifically, the combination arm suffered more grade 3 or higher AEs when compared to the osimertinib arm (80% vs. 52%) at the time of final overall survival analysis. The hallmark toxicity of the MARIPOSA regimen is dermatologic (representing the most common reason for treatment discontinuation), driven by dual *EGFR* inhibition from both amivantamab and lazertinib [23]. Namely, paronychia was the most common adverse event, occurring in 69% of combination-arm patients (with 12% reporting grade 3 AE and above) when compared against 30% of the osimertinib arm reporting the same adverse event. Infusion-related reactions (IRRs) also occurred in 65% of patients receiving amivantamab–lazertinib, with grade 3 or higher AEs in 6%. Moreover, venous thromboembolic events (VTEs) are a distinctive toxicity of amivantamab–lazertinib, with a 40% reported incidence in the combination arm when compared against 11% reported within the monotherapy arm. Other notable adverse effects included but are not limited to hypoalbuminemia, hypocalcemia, muscle spasms, ALT and/or AST elevation, and ILD/pneumonitis. Dose interruptions of any trial agent occurred in 83% of patients, dose reductions in 59%, and discontinuation of any agent in the combination arm in 35% (compared against 39%, 5%, and 14% with osimertinib, respectively) [22].

Given the significantly increased likelihood of multiple adverse effects associated with the different intensification strategies, multiple strategies have been implemented to better manage and mitigate these AEs. One such study was the phase II COCOON trial, which was designed to prospectively evaluate whether an enhanced, prophylactic dermatologic management (DM) regimen could reduce the incidence and severity of patients receiving both amivantamab and lazertinib. The standardized COCOON regimen comprises four different components: (1) oral doxycycline or minocycline twice daily during weeks 1–12, (2) topical 1% clindamycin applied to the scalp daily during weeks 13–52, (3) chlorhexidine 4% wash applied to fingernails and toenails daily, and (4) a ceramide-based non-comedogenic moisturizer applied to the body and face at least once daily. This COCOON DM regimen achieved a striking reduction in the primary endpoint of dermatologic AEs (grade 2 or greater) at 42%, when compared against 75% with the standard-of-care arm (*p* < 0.0001) [82]. Another study, the phase II SKIPPirr trial, sought to systematically evaluate different prophylactic regimens to reduce IRR incidence, with one such regimen involving oral dexamethasone 8 mg twice daily for a total of five doses before the first day of cycle 1. Among the 40 participants treated in this cohort, the cycle 1 day 1 IRR rate was 22.5%, representing an approximately three-fold reduction when compared to the historical rate of 67.4% [83]. Finally, the phase III PALOMA-3 trial evaluated the efficacy and tolerability of a subcutaneous formation of amivantamab. PALOMA-3 enrolled 418 patients with *EGFR*-mutated advanced NSCLC who had progressed after osimertinib and platinum-based chemotherapy, randomizing them either to subcutaneous or intravenous amivantamab, both combined with oral lazertinib. Median administration time for the first dose was reduced from 5 h for IV amivantamab to 4.8 min for subcutaneous amivantamab, with median time-in-chair time similarly being reduced from 6.5 h to 23 min on cycle 1 day 1. Nevertheless, the efficacy of both arms was comparable, with ORRs of 30% and 33% in the subcutaneous and IV groups, respectively. Moreover, IRRs were reduced fivefold (13% vs. 66%), and VTE was lower (9% vs. 14%) in the subcutaneous versus IV group. Most notably, however, overall survival was significantly longer in the subcutaneous vs. the IV cohort (HR 0.62, 95% CI 0.42–0.92, *p* = 0.02) [84]. Taken together, these three studies illustrate a comprehensive, multifaceted strategy that should be considered when optimizing the amivanatmab–lazertinib regimen set forth by MARIPOSA.

Other emerging therapies in NSCLC have also presented unique side effect profiles that require careful monitoring and management. For example, ocular toxicity has become an increasingly recognized side effect of Dato-DXd, with the TROPION-Lung05 study demonstrating that 26.3% of treated patients have reported dry eye, keratitis, blepharitis, increased lacrimation, conjunctivitis, and/or blurry vision [73]. Ivonescimab, a bispecific antibody that targets both *PD-1* and *VEGF* pathways simultaneously, is specifically associated with hemorrhagic toxicity given its inherent antiangiogenic mechanism via *VEGF* blockade. One NSCLC systematic review demonstrated that hemorrhage can be seen in as much as 15.4% of patients using ivonescimab [85]. Finally, savolitinib, a selective MET TKI used in NSCLC patients with EGFR mutations and *MET* overexpression/amplification, is classically associated with peripheral edema, with 46.0% of patients reporting this AE in the SAVANNAH trial [72].

## 5. The Advent of ctDNA

Despite the remarkable efficacy of osimertinib and a multitude of combination regimens in treating advanced *EGFR*-mutant NSCLC as discussed above, the optimal intensity and duration of therapy remain subject to active investigation. Circulating tumor DNA (ctDNA) has emerged as a minimally invasive biomarker with the potential to inform real-time treatment decisions, including both the escalation and de-escalation of therapy. ctDNA refers to fragmented tumor-derived DNA shed from tumors into the systemic circulation. With a short half-life ranging from 16 min to 2.5 h, ctDNA has emerged as a minimally invasive "liquid biopsy" that captures the molecular landscape of a patient’s tumor in real-time [86]. The prognostic value of circulating DNA in cancer was established after a multivariable analysis demonstrated that detection of ctDNA was associated with significantly worse OS (HR 1.91, 95% CI 1.59–2.29, *p* < 0.001) [87]. These findings provided a foundational rationale for incorporating ctDNA quantification into a clinical decision-making framework across numerous tumor types, including NSCLC.

In one of the first studies that addressed ctDNA specifically in the context of *EGFR*-mutant NSCLC, a significant correlation was demonstrated between baseline ctDNA concentration and radiographic tumor burden, such as bone metastasis (odds ratio 3.985, *p* = 0.046) in patients with newly diagnosed advanced-stage disease. Furthermore, PFS was significantly shorter in the group detected with ctDNA and in the undetected ctDNA group (median PFS 9.8 vs. 20.7 months, HR 2.30, 95% CI 1.202–4.385, *p* = 0.012) [88]. These findings underscore the utility of baseline ctDNA quantification as a surrogate for disease volume, and as such, it serves as a prognostic tool at the time of initial treatment selection. However, it is important to note that not all NSCLC tumors shed ctDNA into the circulation. In a study of stage IV EGFR-mutated patients, approximately 35% had undetected EGFR mutations in plasma ctDNA at baseline (“non-shedders”), with shedding status associated with higher ECOG performance status, bilateral tumor localization, and the presence of extrathoracic disease [89].

The clinical utility of serial ctDNA monitoring during EGFR-TKI therapy was rigorously evaluated in exploratory analyses of data drawn from the phase III FLAURA and AURA3 trials. Specifically, plasma *EGFR* mutation (EGFRm) status was measured at baseline and at weeks 3 and 6 of treatment using droplet digital PCR (ddPCR). In AURA3, non-detectable baseline plasma EGFRm had significantly longer median PFS compared to those with detectable mutations (HR 0.48, 95% CI 0.33–0.68, *p* < 0.0001). Among patients with detectable baseline EGFRm, clearance of plasma EGFRm at week 3 was associated with a superior median PFS, with 10.9 months versus 5.7 months with osimertinib, and 6.9 months versus 4.2 months with platinum-pemetrexed. Similarly, in FLAURA, median PFS was longer with non-detectable baseline plasma EGFRm (HR 0.54, 95% CI 0.41–0.70, *p* < 0.0001) when compared against the detectable arm. Among patients with detectable baseline EGFRm, clearance of plasma EGFRm at week 3 also demonstrated a superior median PFS, with 19.8 months versus 11.3 months with osimertinib, and 10.8 versus 7.0 months with either gefitinib or erlotinib [90]. These findings suggest that plasma EGFRm analysis as early as the time of treatment initiation (as well as after three weeks into treatment) has the potential to predict outcomes in advanced *EGFR*-mutated NSCLC. Similar findings were demonstrated when measuring plasma EGFRm in both the monotherapy and combination regimen arms of the FLAURA2 trial, in which baseline non-detectable EGFRm was associated with a higher median PFS compared to baseline detectable EGFRm, and EGFRm clearance at three weeks was associated with a higher median PFS compared to EGFRm non-clearance at three weeks [91].

A longitudinal ctDNA study using data from FLAURA and AURA3 also demonstrated that ctDNA may be able to detect progressive disease (PD) before radiologic detection, as set forth by the RECIST (Response Evaluation Criteria in Solid Tumors) criteria. ctDNA PD preceded or co-occurred with RECIST-defined PD in 64% of FLAURA and 56% of AURA3. More specifically, median time from ctDNA PD to RECIST-defined PD was 3.4 and 2.6 months in the osimertinib and gefitinib/erlotinib arms, respectively (FLAURA), and 2.8 and 1.5 months in the osimertinib and platinum-pemetrexed arms, respectively (AURA3) [92]. These findings further establish the utility that longitudinal ctDNA monitoring may provide in detecting progressive disease in conjunction with standard radiologic methods.

ctDNA dynamics were also evaluated in a secondary analysis of MARIPOSA, which specifically examined outcomes in patients with detectable baseline ctDNA and lack of ctDNA clearance on treatment. The study demonstrated that amivantamab–lazertinib significantly improved median PFS when compared against osimertinib in patients with detectable baseline ctDNA by ddPCR (20.3 vs. 14.8 months, HR 0.68, 95% CI 0.53–0.86, *p* = 0.002). Notably, patients without ctDNA clearance at cycle 3 day 1 derived a particularly pronounced benefit from amivantamab–lazertinib when compared against osimertinib (16.5 vs. 9.1 months, HR 0.48, 95% CI 0.27–0.87, *p* = 0.015), while those with ctDNA clearance also benefited (24.0 vs. 16.5 months, HR 0.74, 95% CI 0.60–0.91, *p* = 0.004) [39]. These findings only further support the clinical value provided by treatment escalation to amivantamab–lazertinib in patients with advanced disease.

Although much of the focus in *EGFR*-mutant NSCLC has been on treatment intensification, ctDNA-guided de-escalation is also an equally important consideration in the management of advanced disease. Specifically, a prospective nonrandomized controlled trial was recently conducted to evaluate adaptive de-escalation of TKI therapy guided by ctDNA in patients with advanced NSCLC (n = 60). Patients with no radiologically detectable disease and undetectable ctDNA after TKI therapy and local consolidative therapy underwent TKI cessation with serial monitoring every 3 months. Of note, treatment was restarted upon either detection of ctDNA, elevated carcinoembryonic antigen (CEA), or radiographic progressive disease. Among the patients who remained in TKI cessation throughout follow-up, the median treatment break duration was 20.3 months. The patients who received retreatment triggered by detectable ctDNA and/or CEA (before radiographic progression) had a median PFS of 20.2 months. Finally, the patients who restarted TKI only after confirmed radiographic progression had a significantly shorter median PFS of 5.5 months. The TKI retreatment response rate was 96%, and the median time to next treatment was 29.3 months [93]. These initial findings suggest that ctDNA-guided adaptive de-escalation is indeed feasible in a subset of advanced *EGFR*-mutant NSCLC patients who have achieved complete remission. These clinical outcomes also support the framework of molecular surveillance in enabling timely treatment before overt clinical progression, preserving treatment efficacy while also providing meaningful drug-free (and thereby adverse event-free) intervals.

The European Organisiation for Research and Treatment of Cancer recently published the APPLE trial, a randomized phase II study that directly tested the feasibility of longitudinal plasma *EGFR* T790M monitoring to guide the order of gefitinib and osimertinib administration in treatment-naive patients. Patients were randomized to three different arms: Arm A (osimertinib upfront as a control cohort), Arm B (gefitinib with switch to osimertinib upon emergence of T790M ctDNA or RECIST progression), and Arm C (gefitinib with switch to osimertinib only at RECIST progression). The primary endpoint was PFS rate on osimertinib at 18 months, which was 67.2% in Arm B, compared against 53.5% in Arm C. Furthermore, the median PFS was 22.0 months in Arm B versus 20.2 months in Arm C, and the median OS was not reached in Arm B versus 42.8 months in Arm C [94]. These findings that are specific to first-generation TKI therapy; however, must be contextualized within the evolving landscape of treating *EGFR*-mutant NSCLC, in which osimertinib or escalation to combination regimens are now the preferred first-line options (as supported by FLAURA2 and MARIPOSA as extensively discussed above). Therefore, future studies should seek to replicate the same ctDNA framework to confirm its utility in monitoring patients on osimertinib-chemotherapy and amivantamab–lazertinib combination regimens [95]. Nevertheless, the APPLE trial has provided essential proof-of-concept evidence that biomarker-driven, ctDNA-guided treatment switching is feasible in identifying patients at risk of progression before radiographic detection.

One key ctDNA study that is currently ongoing is the PACE-LUNG trial, which is designed to evaluate the efficacy and safety of a biomarker-treatment strategy for therapy escalation in EGFR-mutant NSCLC patients at high risk for early treatment failure. Specifically, this multicenter phase II study has been enrolling stage IIIB/IV NSCLC patients with either *EGFR* exon 19 deletion or L858R mutation, who were initiated on first-line osimertinib and underwent ctDNA testing at three to four weeks. Patients with persistent plasma EGFRm ctDNA at this time point would subsequently receive treatment escalation with the addition of four cycles of platinum-pemetrexed, after which osimertinib is then continued as standard of care until disease progression or intolerable toxicity. The primary endpoint of this study is PFS, whereas the secondary endpoints include OS, response rate, safety, and quality of life [96]. Instead of adding chemotherapy to all patients upfront (which increases the likelihood of systemic toxicity and adverse events), PACE-LUNG uses early ctDNA dynamics as a real-time biomarker to selectively escalate therapy only in patients at high risk for early treatment failure. This unique study design will hopefully set a new standard in managing *EGFR*-mutant NSCLC as the treatment framework continues to shift towards the incorporation of ctDNA.

## 6. Discussion

**Figure 2:**
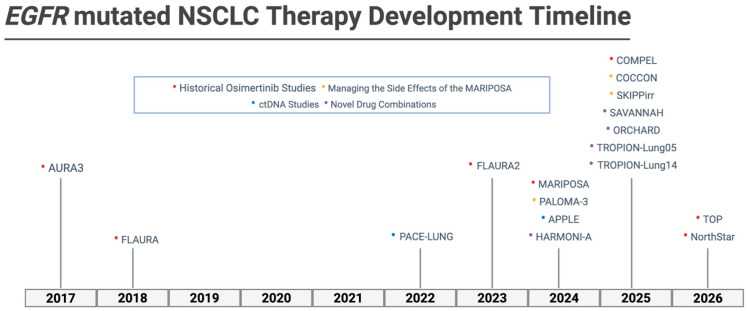
*EGFR*-mutant NSCLC therapy development timeline. (EGFR = epidermal growth factor, NSCLC = non-small cell lung cancer, ctDNA = circulating tumor DNA).

The management of *EGFR*-mutant NSCLC has been rapidly changing, with Table 2 and Figure 2 above demonstrating the evolution from a TKI monotherapy strategy toward a more dynamic, aggressive biomarker and combination regimen-based framework. One central theme emerging from the most recent landmark trials is that this subtype of lung cancer is not a uniform condition, but rather a molecularly heterogeneous disease in which co-occurring gene alterations, including changes in *TP53* and *RB1*, profoundly influence both prognosis and treatment response. This is especially important to consider given the high prevalence of *TP53* co-mutations, the high risk of histologic SCLC transformation in concurrent *RB1* and *TP53* loss, and the consistent association with inferior PFS and OS across multiple studies. Furthermore, the roles of less common co-alterations (including those of *PIK3CA* and *PTEN*) only serve to add further layers of complexity. The clinical outcomes associated with these mutations collectively support an increasingly compelling rationale for universal next-generation sequencing at diagnosis in these patients. Although evaluating for *EGFR* mutations is already considered standard practice, the prognostic value in detecting additional co-alterations argues for broader testing that captures the molecular status of *TP53*, *RB1*, *PIK3CA*, *PTEN*, and *KRAS* at a minimum. Of note, however, since most of these molecular studies were largely retrospective in nature, the possibly predictive value of broader NGS should first be validated with prospective studies and randomized trials.

Of note, the molecular and functional complexity of *TP53* mutations represents a significant limitation of many retrospective studies within the field of NSCLC risk stratification. Most analyses regard *TP53* status as a binary variable (mutant versus wild-type) without accounting for the heterogeneous functional consequences of different *TP53* alterations. Rather, mutations involving the DNA-binding domain, disruptive versus non-disruptive mutations, and exon-specific alterations carry distinct prognostic implications [26,97]. As such, future studies should adopt a more thorough and standardized evaluation of *TP53* mutations to more accurately assess the prognostic and predictive value of such alterations in *EGFR*-mutant NSCLC.

Beyond molecular co-alterations, several clinical and biochemical prognostic variables have been identified in patients with advanced *EGFR*-mutant NSCLC treated with EGFR-TKIs. A retrospective analysis of stage IV patients identified the presence of liver metastasis, the presence of brain metastasis, elevated neuron-specific enolase, and ECOG performance as independent prognostic factors for both PFS and OS across multiple studies [98]. Furthermore, a large multi-institutional analysis (n = 3551) demonstrated that age > 65, male sex, clinical stage IV, and baseline brain metastasis were poor prognostic factors; whereas exon 19 deletion subtype, subsequent T790M development, and osimertinib exposure were associated with favorable outcomes [99]. These clinical prognostic features, when taken together with the molecular features discussed above, should be integrated into comprehensive risk stratification models to guide treatment selection.

Recent and ongoing studies have sought to undermine the central nervous system’s reputation as a “sanctuary site,” and in doing so, they have demonstrated a clear evolution in the management of *EGFR*-mutant NSCLC with CNS involvement. This transition has comprised the inadequate intracranial activity of first-generation TKIs; through the substantial improvement achieved with newer generation TKIs (as set forth by FLAURA and BLOOM for CNS metastasis and LMD, respectively). We have seen further improvements in CNS efficacy with new drugs such as amivantamab, along with combination strategies (as set forth by FLAURA2 and MARIPOSA). Furthermore, the integration of stereotactic radiation therapy, as proposed by the Northstar trial, highlights the potential for multimodal approaches that combine both systemic and local therapies. As such, the optimal sequencing and combination of systemic intensification with radiation remains a critical question that should warrant further prospective investigation, particularly as the field continues to move toward earlier and more aggressive management of CNS disease. Finally, the emerging role of CSF molecular profiling in identifying high-risk patients with CNS involvement also represents a promising frontier. Namely, the association of *CDK4*, *CDK6*, and *MYC* co-alterations in CSF with poorer outcomes provides a biological rationale for risk-stratified approaches to CNS-directed therapy. However, the practical implementation of CSF-based molecular profiling faces multiple challenges, ranging from the low tumor DNA yield in CSF to the inherent risk posed by the lumbar puncture itself [100]. Future studies should address these barriers while also evaluating whether CSF-derived molecular information adds incremental value beyond what can be obtained from plasma ctDNA analysis.

Landmark studies such as the FLAURA2 trial highlight the importance of combining chemotherapy with EGFR-TKIs as first-line treatment in this patient population. Namely, the biological rationale behind this combination regimen rests on the complementary mechanisms of action: EGFR-TKIs target the tumor cell population containing the receptor mutation, while platinum-pemetrexed chemotherapy may eliminate the non-*EGFR*-dependent population and delay the emergence of resistance [20]. The statistically and clinically significant improvement in efficacy demonstrated by the FLAURA2 and MARIPOSA regimens, however, comes at a substantial cost in terms of treatment-related toxicity, and as such, the decision to intensify therapy must be individualized based on a careful assessment of both benefits and risks. Specifically, the observation that grade 3 (or higher) adverse events occurred in significantly higher proportions of the combination therapy arms (when compared to those of the osimertinib monotherapy arms) underscores the magnitude of the incremental toxicity burden. The development of targeted prophylactic strategies to mitigate the toxicities of amivantamab–lazertinib, however, represents a significant advance in supportive care, with COCOON addressing dermatologic AEs, SKIPPirr addressing IRR rates, and PALOMA-3 addressing a multitude of barriers with a subcutaneous reformulation of amivantamab. The positive findings reported by each of these studies now open the door to new avenues that should be explored in future trials. One such avenue concerns the FLAURA2 regimen, in which the side effects of patients on the osimertinib-chemotherapy combination are still typically managed with standard chemotherapy supportive care measures for nausea and cytopenia. However, these patients may benefit from trials similar to the COCOON, SKIPPirr, and PALOMA-3 studies to determine if specific prophylactic measures should be performed before a patient initiates the FLAURA2 regimen to decrease the likelihood of myelosuppression and gastrointestinal toxicity. Furthermore, the advent of subcutaneous amivantamab now raises the possibility of reformulating other agents in future trials to determine if this will either improve clinical efficacy and/or tolerability of regimens in treating EGFR-mutant NSCLC. Finally, the high rates of treatment discontinuation in both combination arms present a clinical challenge: even with effective prophylactic strategies, a substantial proportion of patients are unable to maintain the full intensity of combination therapy. This raises the question of whether intermittent or adaptive dosing strategies, guided by biomarkers such as ctDNA, may preserve efficacy while simultaneously reducing cumulative toxicity. The integration of toxicity management with biomarker-guided treatment adaptation represents a logical next step in optimizing the therapeutic index of various combination regimens.

The emergence of ctDNA as a real-time biomarker in *EGFR*-mutant NSCLC arguably represents the most critical development in assisting with both treatment intensification and de-escalation. Specifically, the data derived from FLAURA, AURA3, FLAURA2, and MARIPOSA consistently demonstrated that both baseline ctDNA status and early ctDNA dynamics (particularly clearance at 3 weeks) are strongly prognostic, with detectable baseline ctDNA and failure to achieve early clearance noted to be associated with significantly inferior PFS across treatment arms. As such, baseline ctDNA quantification may serve as a surrogate for tumor burden that complements radiographic staging in identifying patients at higher risk for early treatment failure, especially given the findings of some studies suggesting that ctDNA may be able to identify these patients before imaging. The concept of ctDNA-guided de-escalation opens an equally important therapeutic avenue, as it has been suggested that drug-free intervals guided by ctDNA do not compromise treatment efficacy (specifically within the APPLE trial). However, the ongoing PACE-LUNG trial design is particularly noteworthy in its pragmatic approach: rather than adding chemotherapy to all patients upfront, it uses early ctDNA dynamics to selectively identify and escalate only those patients at highest risk for early treatment failure. If successful, this approach could substantially reduce the number of patients exposed to the toxicity of combination therapy while simultaneously preserving the efficacy benefits for those who need it most. Other ctDNA studies are currently ongoing, including evaluation of survival measures in patients on osimertinib monotherapy (or osimertinib plus chemotherapy) [101] as well as those on a combination of osimertinib and sacituzumab tirumotecan, a novel TROP2-directed antibody–drug conjugate [102]. Regardless, several challenges must still be properly addressed before ctDNA-guided strategies can be widely implemented. Such areas that must be addressed include standardization of ctDNA assay platforms, thresholds for positivity, and optimal timing of assessment. Furthermore, the outcomes in selecting between ddPCR and NGS-based methods to detect ctDNA should be further evaluated for both clinical utility and cost-effectiveness. There are many different forms of methodologies being used in ctDNA testing, with a large variance in the number of mutations tested, as some ctDNAs do not test for copy number variants, single nucleotide variants, and there are differences in limits of detection [103]. The non-shedding phenotype also continues to pose a fundamental challenge to universal ctDNA-guided treatment algorithms. These non-shedding tumors are more commonly seen among patients with lower disease burden, intrathoracic-only disease, and favorable performance status, and such patients may paradoxically have better outcomes that would not benefit from treatment intensification [104,105]. However, this may limit the clinical utility of a negative ctDNA result for de-escalation decisions. As such, future studies should explicitly report shedding rates and stratify outcomes by shedding status to enable a more accurate interpretation of ctDNA-guided strategies.

Other limitations from the studies discussed should also be considered. First, much of the data supporting risk stratification based on co-mutations derives from retrospective analyses and may not be generalizable across diverse populations. Specifically, the representation of diverse racial and ethnic populations and the landmark trials varies, and the applicability of these findings to underrepresented groups requires further studies. Furthermore, the ctDNA analyses from FLAURA, AURA3, FLAURA2, and MARIPOSA are largely exploratory or secondary endpoints, and prospective validation with dedicated biomarker-driven trials (such as PACE-LUNG) is essential before ctDNA-guided treatment algorithms can be adopted into standard practice. Finally, the follow-up duration for overall survival and progression-free survival in several of the newer trials remains relatively short, and as such, longer-term data are needed to confirm the durability of these observed benefits.

The landscape of *EGFR*-mutant NSCLC management may also benefit from head-to-head comparisons of the FLAURA2 and MARIPOSA regimens, ideally with biomarker-stratified analysis. Moreover, because ctDNA is a non-invasive biomarker that can be sampled repeatedly at different treatment timepoints, there has been an increasing interest in leveraging artificial intelligence (AI) to help transform ctDNA data into models that can guide clinical decision-making [106]. As preliminary studies have begun attempting to incorporate AI into guiding treatment of NSCLC, this may represent another avenue that may be worth further pursuing in future studies, particularly within the *EGFR*-mutant subtype. Finally, patient-reported outcomes and quality-of-life assessments should be systematically incorporated into future trials to ensure that efficacy gains from treatment intensification translate into meaningful clinical benefit from the patient’s perspective.

While ctDNA has emerged as arguably the most clinically advanced biomarker to date, several other technologies are rapidly evolving that may complement, if not enhance, ctDNA-based approaches in EGFR-mutant NSCLC. AI-driven tools are being integrated across multiple domains, including AI-guided digital pathology that can extract quantitative features from histologic images to predict molecular subtypes, treatment response, and prognosis with increasing accuracy [107]. Proteomic profiling of plasma and tumor tissue also provides complementary biomarker discovery avenues; for example, serum protein panels and autoantibody signatures have shown promise for early NSCLC detection and prognostic stratification [108]. Extracellular vesicles (EVs), including exosomes, represent a particularly promising frontier, as tumor-derived EVs carry stable molecular cargo (e.g., proteins, nucleic acids, lipids) that reflect the proteomic, transcriptomic, and metabolic states of parent tumor cells, and, unlike ctDNA, are resistant to nuclease-mediated degradation. Recent proteomic analyses of plasma-derived EVs have identified treatment-induced molecular remodeling signatures that may serve as non-invasive tools for monitoring therapy response and patient stratification [109]. As such, the integration of this technology within an AI-driven analytical framework represents a logical evolution toward a more comprehensive, real-time tumor characterization that extends beyond the genomic information captured by ctDNA alone.

Collectively, the evidence and studies reviewed above underscore a clear yet nuanced trajectory for the field. Namely, the management of *EGFR*-mutant NSCLC is inevitably progressing toward a precision-guided, biomarker-driven paradigm, yet the pace and nature of this transition must be calibrated to account for the realities of clinical practice across diverse healthcare systems. Table 3 synthesizes the major thematic areas discussed in this review into a structured guidance framework encompassing various domains as outlined in this review. The landmark trials discussed throughout this review were largely conducted in high-income settings with broad access to next-generation sequencing, novel biologics such as amivantamab, advanced ctDNA platforms, and specialized supportive care infrastructure. In many regions, however, these resources are not uniformly available or reimbursed. Future clinical guidelines should therefore move toward stratified recommendations that explicitly account for these resource and regional parameters, rather than assuming a single global standard.

## 7. Conclusions

The treatment landscape for EGFR-mutant NSCLC is evolving from a one-size-fits-all approach toward a precision-guided, multifaceted framework that integrates molecular profiling, CNS disease assessment, and dynamic ctDNA monitoring to inform individualized treatment decisions. The evidence derived from multiple landmark trials collectively support a paradigm shift in which: (1) comprehensive genomic profiling at diagnosis identifies patients at higher risk for treatment failure, (2) combination strategies offer meaningful efficacy gains for appropriately selected patients, (3) proactive toxicity management preserves quality of life during treatment intensification, and (4) ctDNA-guided adaptation enables both timely escalation for non-responders and safe de-escalation for patients achieving appropriately molecular responses. As findings from ongoing trials continue to mature, the landscape is poised to move closer to a personalized approach that maximizes therapeutic benefit while minimizing unnecessary treatment burden.

## Figures and Tables

**Table 1:** A selected list of key studies regarding molecular risk factors in *EGFR*-mutant NSCLC. Articles accessed on 19 June 2026. (EGFR = epidermal growth factor receptor, NSCLC = non-small cell lung cancer, TP53 = tumor protein p53, mPFS = median progression-free survival, mOS = median overall survival, DBD = DNA binding domain, RB1 = retinoblastoma 1, SCLC = small cell lung cancer, MET = Mesenchymal-Epithelial Transition, PIK3CA = Phosphatidylinositol-4,5-bisphosphate 3-kinase catalytic subunit alpha, PTEN = Phosphatase and tensin homolog, KRAS = Kirsten rat sarcoma viral oncogene homolog, TKI = tyrosine kinase inhibitor, VAF = variant allele frequency, PD-L1 = Programmed death-ligand 1, TPS = tumor proportion score).

Molecular Alteration	Frequency in *EGFR*-Mutant NSCLC	Prognostic/Predictive Impact
TP53 mutation	55–65%	Shorter mPFS (HR 1.51, 95% CI = 1.33–1.71, *p* = 0.001) and mOS (HR 1.64, CI = 1.33–2.02, *p* = 0.001) compared to absence of TP53 mutation
TP53 mutation subtype (DBD, exon 8, disruptive)	Subset of TP53-mutant cases	DBD mutations and exon 8/disruptive mutations are associated with worse PFS and OS; they facilitate faster acquisition of resistance mutations (e.g., T790M)
RB1 mutation (+TP53)	~5% (concurrent RB1 + TP53)	Poor prognosis; high risk of histological transformation to SCLC; RB1 lost in 100% of SCLC-transformed cases
MET amplification	Observed in ~16% of patients following first-line osimertinib treatment (commonly acquired resistance mechanism)	Typically requires treatment with dual EGFR + MET inhibition (e.g., amivantamab, savolitinib, tepotinib); post-progression molecular testing is essential
PIK3CA mutation	~8%	Poor prognosis, especially when co-occurring with TP53; mOS 39.5 vs. 21.9 months (*p* = 0.046) when compared against wild type
PTEN inactivation	~5–12%	mPFS 10.3 vs. 2.6 months on 3rd-gen TKIs (*p* = 0.001); PTEN loss activates EGFR independently in EGFR-dependent cells
KRAS co-mutation	~1.1%	Rare co-occurrence; may exist in cis or trans configurations; VAF analysis can suggest cis vs. trans relationship
High PD-L1 expression (TPS ≥ 50%)	~10–17%	Strongest predictor of very early progression on 1st-line osimertinib (surpassing TP53); independently predicts shorter survival

**Table 2:** A selected list of key clinical trial results in treating EGFR-mutant NSCLC. Articles accessed on 22 April 2026. (EGFR = epidermal growth factor receptor, NSCLC = non-small cell lung cancer, mPFS = median progression-free survival, HR = hazard ratio, mOS = median overall survival, ORR = objective response rate, TKI = tyrosine kinase inhibitor, ECOG = Eastern Cooperative Oncology Group, CNS = central nervous system, AE = adverse event, IRR = infusion-related reaction, BID = bis in die (twice daily), C1D1 = cycle 1 day 1, RECIST = Response Evaluation Criteria in Solid Tumors, PD = progressive disease, ctDNA = circulating tumor DNA, mDOR = median duration of response, Dato-DXd = datopotamab deruxtecan, DCR = disease control rate, mg = milligram, kg = kilogram).

Trial Name	Trial Number and Phase	Study Design	Target Population	Key Results
Historical Osimertinib Studies				
AURA3	NCT02151981, phase III (n = 419)	Osimertinib vs. platinum-pemetrexed	Chemotherapy-naive patients with *EGFR* T790M-positive advanced NSCLC progressing on prior EGFR-TKI	mPFS 10.1 vs. 4.1 mo (HR 0.30, *p* < 0.001) mOS 26.8 vs. 22.5 months (HR 0.87, *p* = 0.277) ORR 71% vs. 31% (odds ratio 5.39, *p* < 0.001)
FLAURA	NCT02296125, phase III (n = 556)	Osimertinib vs. 1st generation TKI (gefitinib/erlotinib)	Patients with previously untreated *EGFR*-mutated (ex19del/L858R) advanced NSCLC	mPFS 18.9 vs. 10.2 mo (HR 0.46, *p* < 0.001) mOS 38.6 vs. 31.8 mo (HR 0.80, *p* = 0.046) ORR 80% vs. 76% (odds ratio 1.27, *p* = 0.24)
FLAURA2	NCT04035486, phase III (n = 557)	Osimertinib + platinum-pemetrexed vs. osimertinib monotherapy	Patients with previously untreated *EGFR*-mutated advanced NSCLC	mPFS 25.5 vs. 16.7 mo (HR 0.62, *p* < 0.001) mOS 47.5 vs. 37.6 mo (HR 0.77, *p* = 0.02) CNS metastasis subgroup: mPFS 24.9 vs. 13.8 mo (HR 0.47)
MARIPOSA	NCT04487080, phase III (n = 1074)	Amivantamab + lazertinib vs. osimertinib monotherapy	Patients with previously untreated *EGFR*-mutated advanced NSCLC	mPFS 23.7 vs. 16.6 mo (HR 0.70, *p* < 0.001) 3-year OS 60% vs. 51% (HR 0.75, *p* = 0.005) CNS metastasis subgroup: mPFS 18.3 vs. 13.0 mo (HR 0.69)
COMPEL	NCT04765059, phase III (n = 98)	Osimertinib + platinum-based chemotherapy vs. placebo + platinum-based chemotherapy	Patients with *EGFR*-mutated advanced NSCLC following non-CNS progression on osimertinib	mPFS 8.4 vs. 4.4 mo (HR 0.43) mOS 15.9 vs. 9.8 mo (HR 0.71) CNS metastasis subgroup: mPFS 15.9 vs. 8.6 mo (HR 0.56)
Northstar	NCT03667820, phase II (n = 42)	Osimertinib for 8 weeks, followed by consolidative radiation therapy to persisting lesions, followed by continued osimertinib until progression or intolerance	Patients with *EGFR*-mutated advanced NSCLC with stable or responding disease after 8 weeks of osimertinib 80 mg	mPFS 32.3 mo (95% CI 21.9–51.7) mOS 45 mo (95% 39.3–56.4 mo) Median duration of osimertinib 32.4 months
TOP	NCT04695925 (n = 294)	Osimertinib + carboplatin-pemetrexed vs. osimertinib monotherapy	Patients with *EGFR*-mutated advanced NSCLC with *TP53*-comutation with ECOG 0 or 1	mPFS 34.0 vs. 15.6 (HR 0.44, *p* < 0.001)
Managing the Side Effects of the MARIPOSA Regimen				
COCOON	NCT06120140, phase II (n = 201)	Enhanced dermatologic management vs. standard of care dermatologic management	Patients with locally advanced or metastatic *EGFR*-mutated NSCLC treated with amivantamab + lazertinib	Grade 2+ dermatologic AEs by week 12: 42% vs. 75% (odds ratio 0.24, *p* < 0.0001)
SKIPPirr	NCT05663866, phase II (n = 68)	4 IRR prophylactic approaches tested: dexamethasone 4 mg (2 doses), dexamethasone 8 mg BID (5 doses), montelukast, subcutaneous methotrexate	Patients with *EGFR*-mutated NSCLC receiving amivantamab + lazertinib after progression on osimertinib and platinum-based chemotherapy	C1D1 IRR rate of 22.5% with dexamethasone 8 mg BID (5 doses), an approximately three-fold reduction when compared to the historical rate of 67.4%
PALOMA-3	NCT05388669, phase III (n = 418)	Oral lazertinib + subcutaneous amivantamab vs. oral lazertinib + intravenous amivantamab	Patients with *EGFR*-mutated advanced or metastatic NSCLC following progression on osimertinib and platinum-based chemotherapy	ORR 30% vs. 33% IRR 13% vs. 66% OS significantly longer in the subcutaneous vs. intravenous group (HR 0.62, *p* = 0.02)
ctDNA Studies				
APPLE	NCT02856893, phase II (n = 156)	Osimertinib upfront (arm A) vs. gefitinib with switch to osimertinib at ctDNA T790M detection or RECIST PD (arm B) vs. gefitinib with switch to osimertinib at RECIST PD only	Treatment-naive patients with *EGFR*-mutated advanced NSCLC	ctDNA T790M monitoring was feasible; 17% of Arm B patients switched to osimertinib based on molecular progression before RECIST PD
PACE-LUNG	NCT05281406, phase II	Treatment with additional platinum/pemetrexed chemotherapy (4 cycles) followed by continuation of osimertinib	Patients with persistent plasma ctDNA *EGFR* mutations at 3–4 weeks after starting osimertinib	Trial currently underway to analyze PFS, OS, and mutational evolution in ctDNA upon disease progression
Novel Drug Combinations				
SAVANNAH	NCT03778229, phase II (n = 365)	Treatment with savolitinib + osimertinib	Patients with *EGFR*-mutated advanced NSCLC and MET amplification following progression on osimertinib	ORR 56.3%, mDOR 7.1 mo, mPFS 7.4 mo
ORCHARD	NCT03944772, phase II (n = 69)	Treatment with osimertinib + Dato-DXd	Patients with *EGFR*-mutated advanced NSCLC progressing on osimertinib	At 4 mg/kg: ORR 43%, mPFS 9.5 mo, mDOR 6.3 mo, mOS 19.8 mo At 6 mg/kg: ORR 36%, mPFS 11.7 mo, mDOR 20.5 mo, mOS 26.2 mo
HARMONI-A	NCT05184712, phase III (n = 322)	Ivonescimab + chemotherapy vs. placebo + chemotherapy	Patients with *EGFR*-mutated advanced NSCLC after progressing on EGFR-TKI	mPFS 7.1 vs. 4.8 mo (HR 0.46, *p* < 0.001) ORR 50.6% vs. 35.4% (*p* = 0.006)
TROPION- Lung05	NCT04484142, phase II (n = 137)	Treatment with Dato-DXd 6 mg/kg	Patients with advanced NSCLC who progressed on targeted therapy and platinum-based chemotherapy	Within the *EGFR*-mutated subgroup: ORR 43.6%, mDOR 7.0 mo, overall DCR 82.1%, mPFS 5.8 mo
TROPION- Lung14	NCT06350097, phase III (n = 562)	Osimertinib + Dato-DXd vs. osimertinib monotherapy	Patients with *EGFR*-mutated locally advanced or metastatic non-squamous NSCLC	Currently enrolling patients; primary endpoint of PFS, with secondary endpoints of OS and DOR

**Table 3:** A guidance framework for future research and treatment in *EGFR*-mutant NSCLC (*EGFR* = epidermal growth factor receptor, NSCLC = non-small cell lung cancer, NGS = next generation sequencing, *TP53* = tumor protein p53, *RB1* = retinoblastoma 1, LMD = leptomeningeal disease, CSF = cerebrospinal fluid, SRT = stereotactic radiotherapy, WBRT = whole-brain radiation therapy, ctDNA = circulating tumor DNA, IRR = infusion-related reaction, ddPCR = droplet digital PCR).

Domain of *EGFR*-Mutant NSCLC Management	Future Research Priorities	Treatment Considerations	Region and Resource Specific Considerations
Molecular risk stratification and NGS	Head-to-head comparison of osimertinib monotherapy vs. combination therapy in molecularly defined high-risk subgroups	• Universal NGS at the time of diagnosis is recommended for all patients • Consider osimertinib and platinum-pemetrexed for *TP53* co-mutation • Consider osimertinib and platinum/etoposide for concurrent *TP53* + *RB1*	• Access to broad NGS may be limited in low/middle-income populations (a selected panel of co-mutations may serve as a cost-effective surrogate) • Platinum-pemetrexed is more widely accessible than biologic therapies globally, and should thus be prioritized where biologic therapies are unavailable or not reimbursed
Brain metastases and LMD	Prospective trials comparing optimal sequencing of systemic therapy and SRT for metastatic CNS disease	• Asymptomatic or minimally symptomatic CNS disease: osimertinib monotherapy remains a standard option (FLAURA) • Active/symptomatic CNS metastases: consider upfront osimertinib (FLAURA2) + platinum-pemetrexed or amivantamab-lazertinib (MARIPOSA) • LMD: High-dose osimertinib (BLOOM) with consideration of SRT for selected symptomatic lesions • Multidisciplinary input (neuro-oncology, radiation oncology) is recommended for all cases	• WBRT may be considered if SRT availability is unavailable • Amivantamab-lazertinib may not be available in many regions; osimertinib + chemotherapy may be a more accessible intensification strategy • CSF profiling is resource-intensive and not broadly feasible outside many academic centers; plasma ctDNA may partially substitute if not available
First-line treatment intensification	Head-to-head randomized trial comparing FLAURA2 regimen vs. MARPIOSA regimen vs. osimertinib monotherapy with biomarker stratification	• Low-risk (no co-mutations, no CNS disease, low ctDNA burden): osimertinib monotherapy remains appropriate • High-risk (co-mutation, CNS metastases, high ctDNA burden): upfront intensification preferred (osimertinib + platinum-pemetrexed or amivantamab-lazertinib)	• Amivantamab-lazertinib may not be available in many regions; osimertinib + chemotherapy may be a more accessible intensification strategy • Subcutaneous amivantamab significantly reduces chair time and IRR, which may be beneficial where IV infusion resources or clinic capacity are limited • Population differences in *EGFR*-mutation prevalence (10–15% Western vs. 40–50% East Asian) may affect the generalized trial results; local epidemiologic context should be considered
Toxicity management and supportive care	Dedicated prophylactic toxicity trials for the FLAURA2 regimen (analogous to trials for the MARIPOSA regimen) to reduce myelosuppression and GI toxicity	• Implement COCOON prophylaxis to mitigate dermatologic toxicity as able • Implement dexamethasone pre-cycle 1 (SKIPPirr trial) • Subcutaneous amivantamab preferred when available (PALOMA-3; 5-fold reduction in IRR, reduced chair time)	• Prophylactic doxycycline or minocycline and topical agents (COCOON) may not be available or reimbursed in all regions • As such, local antibiotic alternatives should be evaluated within such regions
Escalation and de-escalation guided by ctDNA dynamics	Complete prospective validation of ctDNA-guided escalation (PACE-LUNG) and de-escalation in *EGFR*-mutant NSCLC	• Standardize ctDNA assay platforms, clearance thresholds, and optimal assessment timepoints (baseline, week 3, week 6) • ctDNA clearance at week 3 suggests a favorable prognosis (may support de-escalation strategies) • If persistent ctDNA at week 3–6 on osimertinib, the patient may be a candidate for escalation (via PACE-LUNG strategy)	• ctDNA assays (especially NGS-based) are expensive and not reimbursed in many low-income populations; ddPCR-based plasma *EGFR* mutation testing is more accessible and cost-effective • Serial ctDNA monitoring requires infrastructure for repeated blood sampling and rapid turnaround (may not be feasible in rural settings)
Novel agents and combinations in the post-osimertinib setting	Further validation of Dato-DXd + osimertinib, ivonescimab, and BH-30643 as intensification strategies via higher-phase trials	All emerging therapies should be considered within the context of prior lines and molecular profiles	• Most novel agents are not yet approved, thereby globally limiting access • Enrolling patients from diverse geographic/ethnic backgrounds in ongoing trials is essential to ensure global applicability of results

## Data Availability

No new data was created or analyzed in this study.
